# Ultrasensitive electrochemical dipstick lateral flow immunoassay for extracellular vesicle quantification using starch-iodine functionalized gold nanoparticles

**DOI:** 10.1007/s00604-026-08063-x

**Published:** 2026-04-22

**Authors:** Clara Saweres-Argüelles, Alberto Sánchez-Calvo, Esther Serrano-Pertierra, Gemma Gutiérrez, María Matos, María Carmen Blanco-López

**Affiliations:** 1 Department of Physical and Analytical Chemistry & Institute of Biotechnology of Asturias, Faculty of Chemistry, University of Oviedo, C/Julián Clavería 8, Oviedo, 33006 Spain; 2 Department of Biochemistry & Institute of Biotechnology of Asturias, University of Oviedo, Santiago Gascón building, C/Julián Clavería s/n, Oviedo, 33006 Spain; 3 Department of Chemical and Environmental Engineering, Institute of Biotechnology of Asturias, Faculty of Chemistry, University of Oviedo, C/Julián Clavería 8, Oviedo, 33006 Spain

**Keywords:** Electrochemical lateral flow immunoassay, Extracellular vesicles, Starch-iodine complex, Biomarkers, Point-of-care

## Abstract

Extracellular vesicles (EVs) have emerged as promising biomarkers for the diagnosis and monitoring of various diseases; however, their clinical application is limited by the need for sensitive and accessible detection platforms. In this study, a highly sensitive electrochemical lateral flow immunoassay (eLFIA) for the detection and accurate quantification of EVs in human plasma is presented. Using a polymer precipitation-based kit, EVs were isolated and determined by using iodine-doped starch-functionalized gold nanoparticle based immunochromatographic test. This strategy has never been used before for electrochemical coupling and it has been protected by an international patent. With this electrochemical approach, the biosensor achieved a detection limit of 9 EVs/µL, demonstrating significantly higher sensitivity than conventional optical methods and even exceeding the limits reported by more complex bulk widely-used techniques, such as Nanoparticle Tracking Analysis. In addition, validation on real biological samples confirmed the accuracy of the device with recoveries above 98%, underlining its robustness and reproducibility. This work establishes a versatile and efficient platform for EV detection that addresses key limitations of current immunoassays, positioning this eLFIA as a promising tool for clinical diagnostics. Furthermore, the proposed strategy has the potential to be adapted to other biomarkers, broadening its applicability to a variety of pathologies.

**Graphical Abstract:**

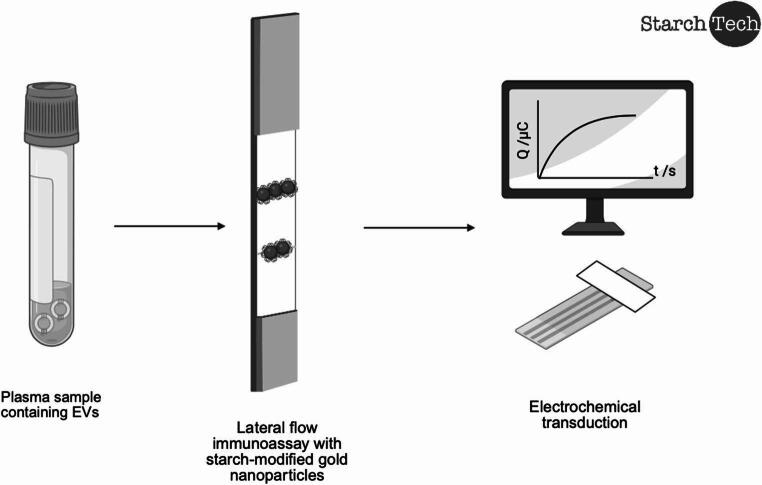

**Supplementary Information:**

The online version contains supplementary material available at 10.1007/s00604-026-08063-x.

## Introduction

Bidirectional communication between cells and their environment is essential for the maintenance of normal physiological processes and plays a crucial role in pathological conditions. Over the past decade, extracellular vesicles (EVs) have emerged as key mediators of intercellular communication. EVs are nano- to micrometre-sized particles surrounded by a lipid bilayer membrane that are released by virtually all cell types [1, 2]. They are therefore present in almost all tissues and body fluids. Their unique ability to transport bioactive molecules underlines their central role in the regulation of cellular functions and their involvement in a wide range of diseases [3]. Thus, EVs have been identified as promising non-invasive biomarkers in several conditions such as cancer [4, 5] and cardiovascular disease [6], reflecting pathological changes through their abundance and specific cargo, opening new opportunities for early diagnosis and monitoring of disease progression.

Accurate detection of EVs presents unique challenges because they are heterogeneous in size, origin, and molecular composition. Despite this heterogeneity and the fact that no universal biomarker has been identified for all EVs, several protein families are expressed recurrently. In particular, membrane-associated proteins, tetraspanins, such as CD9, CD63 or CD81, together with cytosolic proteins as TSG101 or ALIX [1]. Furthermore, as mentioned above, they are present in all types of body fluids with a variety of other cellular and molecular components that can interfere with analytical results [7]. Traditionally, the most commonly used techniques for EV detection include ultracentrifugation, which is considered the reference standard, microscopy-based methods, flow cytometry and enzyme-linked immunosorbent assays (ELISA) [8]. Although these methods provide reliable results, they have important limitations such as technical complexity, high cost and, in the case of immunoassays, low sensitivity, which is insufficient to detect the low concentrations of EVs present in biological fluids, especially in the early stages of disease.

In response to these challenges, innovative methods for EV isolation and detection have recently been developed based on technologies such as microfluidics, biomolecular probes, nanomaterials, surface plasmons and magnetic technology [9, 10]. These advanced strategies have brought the possibility of implementing liquid biopsies based on EV detection much closer, with great potential to transform the early and non-invasive diagnosis of various diseases. However, many of these methods still face significant barriers to their routine use in clinical settings. These limitations include low throughput and the need for signal amplification, which complicate their implementation in widely used diagnostic scenarios. In this sense, ultra-sensistive biosensing strategies have been reported to simplify analytical protocols, improving detection performance, including electrochemical immunosensors, easy to adjust to clinical settings, and washing-free sensing approaches, designed to reduce sample manipulation while keeping a high sensitivity [11–13].

In this context, lateral flow immunoassays (LFIA) have emerged as a promising solution, combining portability, speed and simplicity [14]. These tests are a particularly suitable alternative for point-of-care diagnostics, as demonstrated in classic applications such as pregnancy testing and, more recently, in the mass diagnosis of viral diseases during the SARS-CoV-2 pandemic. However, the limited sensitivity of conventional optical formats reduces their applicability in the detection of biomarkers present at extremely low concentrations, such as in the case of certain EV biomarkers. To overcome this limitation, the incorporation of electrochemical methods into LFIA has emerged as an innovative strategy capable of significantly improving their sensitivity [15], thus expanding their potential in detection applications.

A variety of labels can be used in electrochemical lateral flow immunoassays (eLFIA). However, although gold nanoparticles (Au NPs) are the most commonly used label in visual analysis of LFIA, their use in eLFIA has been limited. When they are used, methods that involve dissolving the particles with acid [16] in the test line or enzymatic digestion [17] prior to electrochemical measurement are often employed.

Functionalization of Au NPs for use in LFIA begins with their bioconjugation to the detection antibody used, followed by blocking free sites on the NPs where no antibody has bound to prevent non-specific adsorption on the test strip. Proteins such as bovine serum albumin (BSA) or casein and polymers are often used for this purpose [18]. In this context, starch is an ideal polymer as it not only blocks the binding sites, thus avoiding possible false positives, but could also act as a functional carrier for an electrochemical probe, such as iodine, due to the known stable complex formed by these two compounds, where iodide ions are introduced into the amylose helix of starch [19]. Although a recent study has explored the use of iodine chemistry for gold measurement by electrochemical redissolution of NPs in the test line [20], our strategy minimizes experimental errors associated with downstream manipulations such as immersion time or reagent concentration. This approach improves significantly the reproducibility of the assay and provides a solid and robust platform that could be used by non-experts.

This study presents the development of a highly sensitive eLFIA specifically designed for the accurate detection and quantification of EV biomarkers. This system is based on the functionalization of gold nanoparticles with starch and iodine, a novelty that not only improves the chemical stability of the assay but also enables electrochemical signal detection at low concentration ranges, overcoming the inherent limitations of conventional optical formats. The proposed approach addresses the key challenges of current immunosensors, such as low sensitivity and the need for complex protocols, by offering a platform that combines simplicity of operation with high analytical performance. Its design, validated with real biological samples, has been shown to be robust and accurate. These features make this biosensor a promising tool for the early and non-invasive diagnosis of various pathologies, with the potential to be adapted for the detection of other biomarkers of interest in EVs.

## Materials and methods

### Isolation of EVs from human plasma

Plasma samples from healthy donors were centrifuged at 3200 × g for 20 min to obtain platelet-poor plasma (PPP). They were then centrifuged a second time at 10,000 × g for 30 min. Plasma-derived EVs were isolated using ExoQuick precipitation reagent. Samples were initially treated with 5 U/mL thrombin and incubated with ExoQuick for 1 h at 4 °C. EV-enriched fractions were collected by centrifugation at 1500 × g for 30 min and resuspended in HEPES.

Particle number and size distribution of the obtained fractions were analyzed by NTA at Nanovex Biotechnologies (Asturias, Spain).

### Synthesis of the starch-iodine complex

Preliminary experiments with several types of starches (amaranth, quinoa and rice) containing different amylose/amylopectin ratios were performed to optimize the complexation of starch-iodine. A 1 mg/mL solution of native quinoa starch (reported to contain 20.95% amylose and 79.05% amylopectin) [21] in milliQ water and heated at 80 °C for 30 min was prepared. Then, Lugol reagent (5% I_2_ and 10% KI solution) was added and shaken, followed by the addition of absolute ethanol to precipitate the coloured starch. The sample was centrifuged at 15,880 x g for 4 min The supernatant was discarded, and the pellet was resuspended in phosphate buffer (PB) up to the initial volume of the solution.

### Bioconjugation of Au NPs

A solution of 1.5 mL of 40 nm Au NPs was carefully mixed with 100 µL of a solution of 0.15 mg/mL of anti-CD63, used as a detection antibody. After 1 h of reaction, 100 µL of a blocking solution containing a 0.1% of iodine-doped quinoa starch was added. The mixture was incubated for 45 min with constant agitation. The nanoparticles were then centrifuged at 9184 x g for 20 min and, after discarding the supernatant, resuspended in 250 µL of 2 mM phosphate stabilizing buffer at pH 7.4 with a concentration of 1% BSA and 10% sucrose. For comparison, the same procedure was carried out using 0.1% BSA as a blocking agent. This procedure is protected under patent [22].

### Preparation of the strips

The LFIA was performed in a dipstick format. The test strips contained a sample pad, a nitrocellulose membrane, an absorbent pad and a plastic base. First, the 25 mm wide nitrocellulose membranes were attached to the plastic base to achieve a stable system. An anti-CD63 test line and an anti-IgG control line were then applied across the membrane using a dispenser at a rate of 0.100 µL/mm. The membrane was then dried at 37 °C for 30 min. Finally, the sample and absorbent pads were adhered to the base with an overlap of 2 mm. The entire card was cut into 5 mm wide strips for subsequent individual assays.

### LFIA

Nanoparticles blocked with iodine-doped starch were tested as labels in lateral flow assays using anti-CD9 as a capture probe and anti-CD63 as a detection antibody. The assay was performed by adding 10 µL of the previously conjugated gold nanoparticles (either blocked with iodine-doped starch or BSA) and 5µL of the corresponding sample (with the desired EVs concentration) to microcentrifuge tubes containing running buffer containing 1% BSA, 0.05% Tween 20 and 150 mM NaCl in HEPES up to a total final volume of 100 µL. The strips were placed vertically and allowed to run for 20 min.

### Electrochemical measurement

**Scheme 1:**
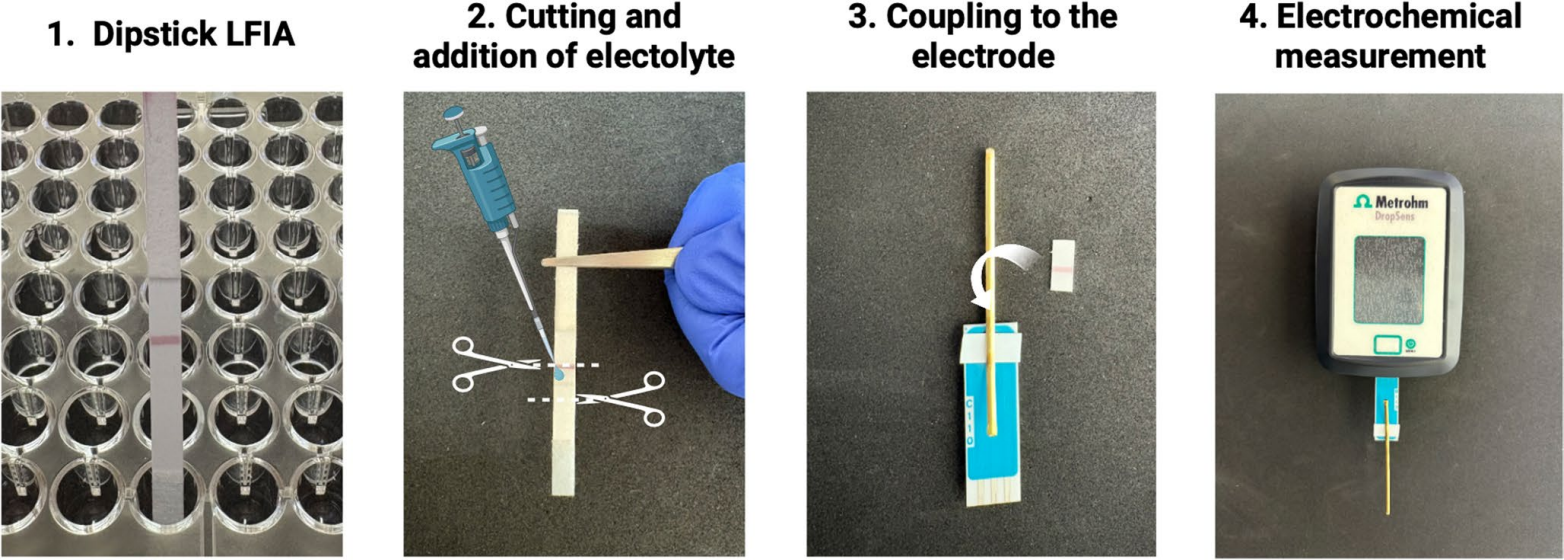
Electrochemical set-up diagram for the measurement of the strip. After the dipstick LFIA is performed, the detection area is cut, and the background electrolyte is added. With the help of a bobby pin, the strip facing downwards is placed on top of the electrode and the coulometry can be performed connecting the electrode to a potentiostat

As shown in Scheme 1, at the end of the test, the test line region was placed in a microcentrifuge tube containing 300 µL PBST (0.01 M + 1% Tween20) for 2 min to remove non-specific adsorption over the strip. It was then dried with paper and prepared for the screen-printed electrode (SPE) platform. Next, 5 µL of 0.5 M sulfuric acid was added to the membrane as an inert electrolyte. This was then coupled to the electrode with the help of a bobby pin, and additions of sulfuric acid were made to the auxiliary and reference electrodes respectively, so that they are not dry. Finally, the electrochemical measurement by coulometry was performed by applying a fixed potential of + 0.45 V for either 90 s (for low concentrations of EVs) or 180 s (for the higher concentrations). This technology has been protected with an international patent [22] and registered as Starch Tech™.

## Results and discussion

### Principle of the lateral flow immunoassay for EVs

The immunoassay used in this study is based on a sandwich format with two anti-tetraspanin antibodies, αCD9 for capture and αCD63 for detection, based on previous results of the research group [23, 24]. These two proteins are present in the membrane of most EVs and can therefore be considered as generic biomarkers of EVs.

**Fig. 1:**
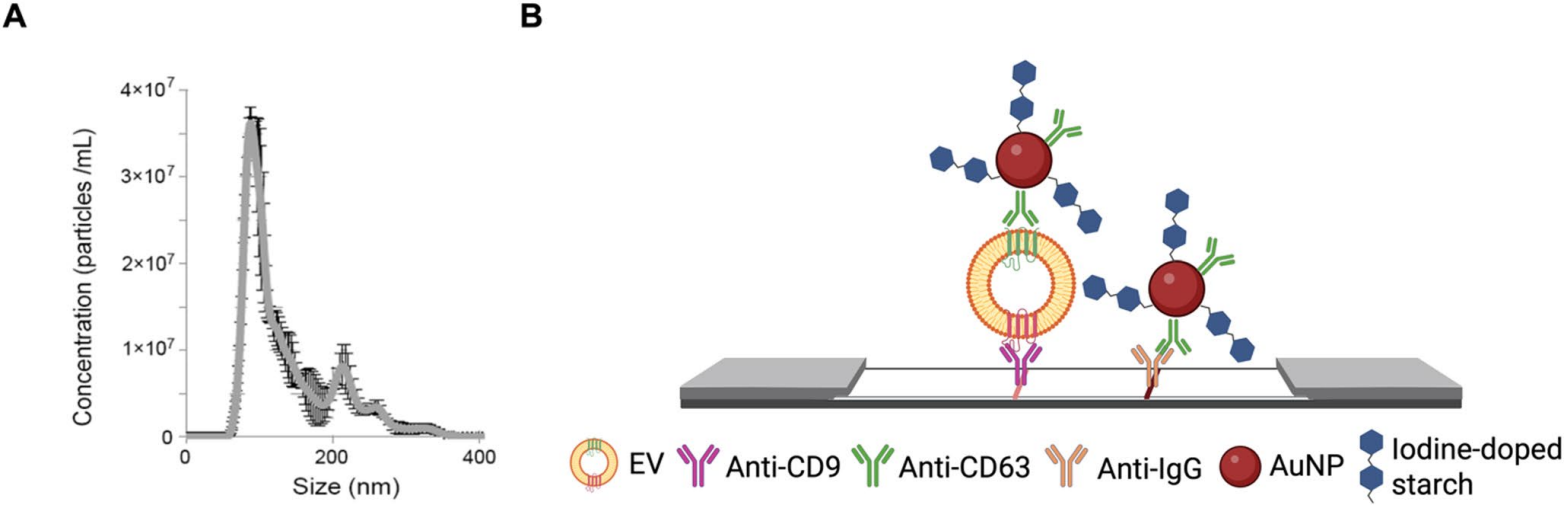
(**A**) NTA size distribution profile of plasma-derived EVs with a mean of 117 nm and a mode of 92 nm. (**B**) Schematic illustration of the LFIA (side view) based on Au NPs as reporter labels blocked with iodine-doped quinoa starch for the detection of EVs

The EV samples from real human plasma were diluted in a buffer solution to the desired concentration ranges, according to the results obtained by nanoparticle tracking analysis (NTA) (Fig. 1A). This would serve to construct the calibration curve of the developed method (9 × 10^9^ − 1 × 10^11^ and 5.4 × 10^4^−2.7 × 10^5^ EV/mL). To perform the test (in dipstick format), the strip was placed in a microcentrifuge tube containing the AuNPs, the EVs sample and the running buffer, which in all cases consisted of 1% BSA, 0.05% Tween20, 150 mM NaCl in HEPES pH 7.4.

The complex formed by the Au NPs with the EV-CD63^+^ EVs thus flowed along the strip by capillary action and was retained after recognition of these EVs (also CD9^+^) by the αCD9 present in the test line (Fig. 1B). The excess of this complex was retained by affinity on the control line printed with anti-mouse IgG immunoglobulin.

### Evaluation of starch as a stabilizer and optimization of the immunoassay

**Fig. 2:**
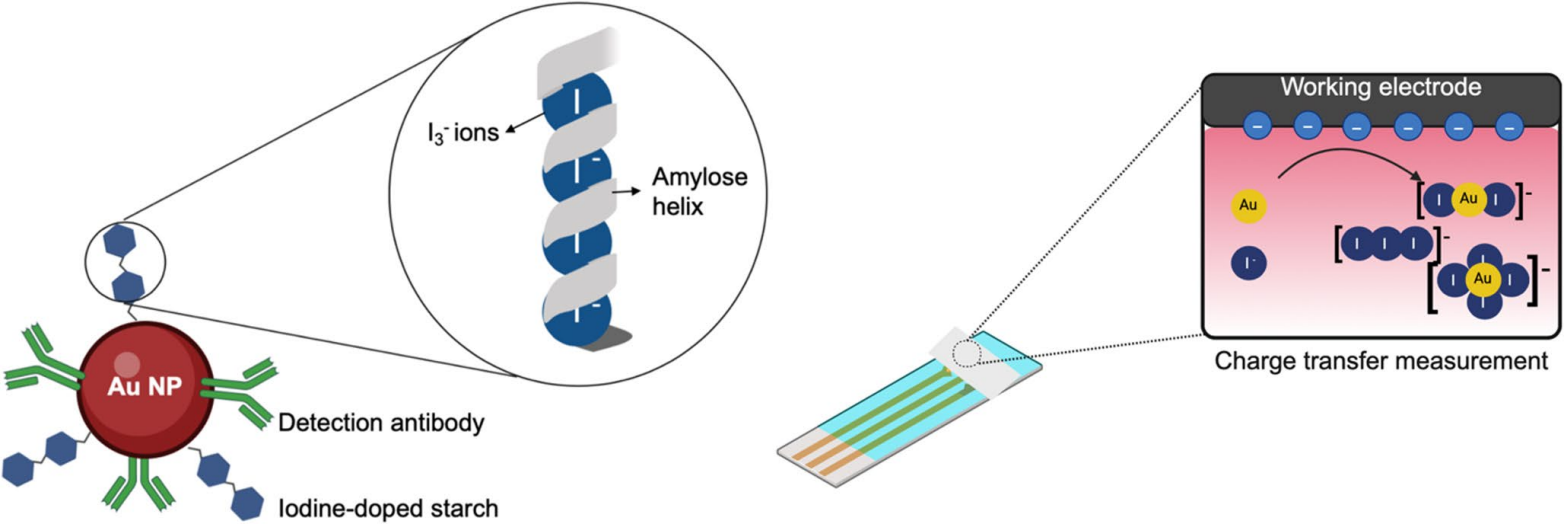
Scheme of Au NP blockage and the reactions taking place for the electrochemical measurement. Enlarged is the schematic of the starch-iodine complex, in which the I_3_^−^ ions are embedded in the amylose helix that forms starch together with amylopectin

Commercially available Au NPs were functionalized with EV-specific antibodies by direct adsorption, taking advantage of the high affinity of the antibodies towards the gold surface [25]. In this bioconjugation process, we propose the use of starch [26], a renewable, biodegradable, biocompatible and inexpensive natural polymer, as a blocking agent and/or functional material that would avoid unspecific interactions. This was based on the properties of starch, in particular its ability to form a unique and stable blue complex with iodine, which could be used as a potential electrochemical probe. Hence, these particles would provide functional advantages by integrating the blocking ability of NPs and electrochemical sensing, as discussed below and shown in Fig. 2.

As for the starch-iodine complex mentioned above, it is formed by the insertion of polyiodides (I_n_^−^), mostly triiodide, into the hydrophobic region of the amylose helix, one of the main components of this polysaccharide [19, 27]. Hence, a starch with a high amylose content was sought. Therefore, starches with different amylose contents were evaluated, including rice, amaranth and quinoa [21]. The resulting complexes showed different colors and UV-Vis absorption spectra depending on the kind of starch (Figure S1A). Rice starch was immediately discarded as it generated a greyish suspension with visible precipitation, indicating a poor complex stability. Amaranth starch formed a reddish complex with a maximum wavelength (λ_max_) around 524 nm, while quinoa starch, with reportedly a similar percentage of amylose, formed a characteristic blue complex with λ_max_ at 614 nm. These differences are related to the length and organization of the present glucan chains, which determine the polyiodide species that they can accommodate within the helices [28]. As quinoa starch produced the desirable blue complex without aggregation, it was selected for the subsequent experiments.

The amount of starch required for the functionalization of the Au NPs was also tested by trying two different concentrations: 1 mg/mL and 5 mg/mL, which are standard concentrations for this type of blocking. However, when the resulting particles were analyzed by dynamic light scattering (DLS), slight aggregation corresponding to unbound starch was observed in the case of the particles with a higher starch concentration (Figure S1B), and a higher polydispersity index was obtained: 0.267 ± 0.006 for the higher concentration and 0.198 ± 0.004 for the lower concentration.

On the other hand, a flocculation test (Fig. S2) was also necessary to determine the optimal detection antibody concentration required to stabilize the Au NPs while avoiding saturation of the surface. This would generate steric hindrance and result in a loss of bioconjugate functionality [29]. To do this, increasing αCD63 concentrations (0–0.2.2 mg/mL) were incubated with the Au NPs prior to salt addition (10% NaCl). When the Au NPs do not have enough antibody coverage, they aggregate producing a visible change of color from red to purple due to the localized surface plasmon resonance (LSPR) effect. As the antibody concentration increased, the NPs remained stable, keeping the characteristic red color of 40-nm Au NPs. The second-lowest concentration of antibodies that showed no aggregation, 0.15 mg/mL, was selected for the development of the immunoassay.

### Characterization of the conjugates

To test the success of the blocking and the stability of the nanoparticles with this blocking complex, Au NPs were bioconjugated with αCD63 and blocked with the synthesized iodine-doped starch complex and with BSA, used as a standard control.

**Fig. 3:**
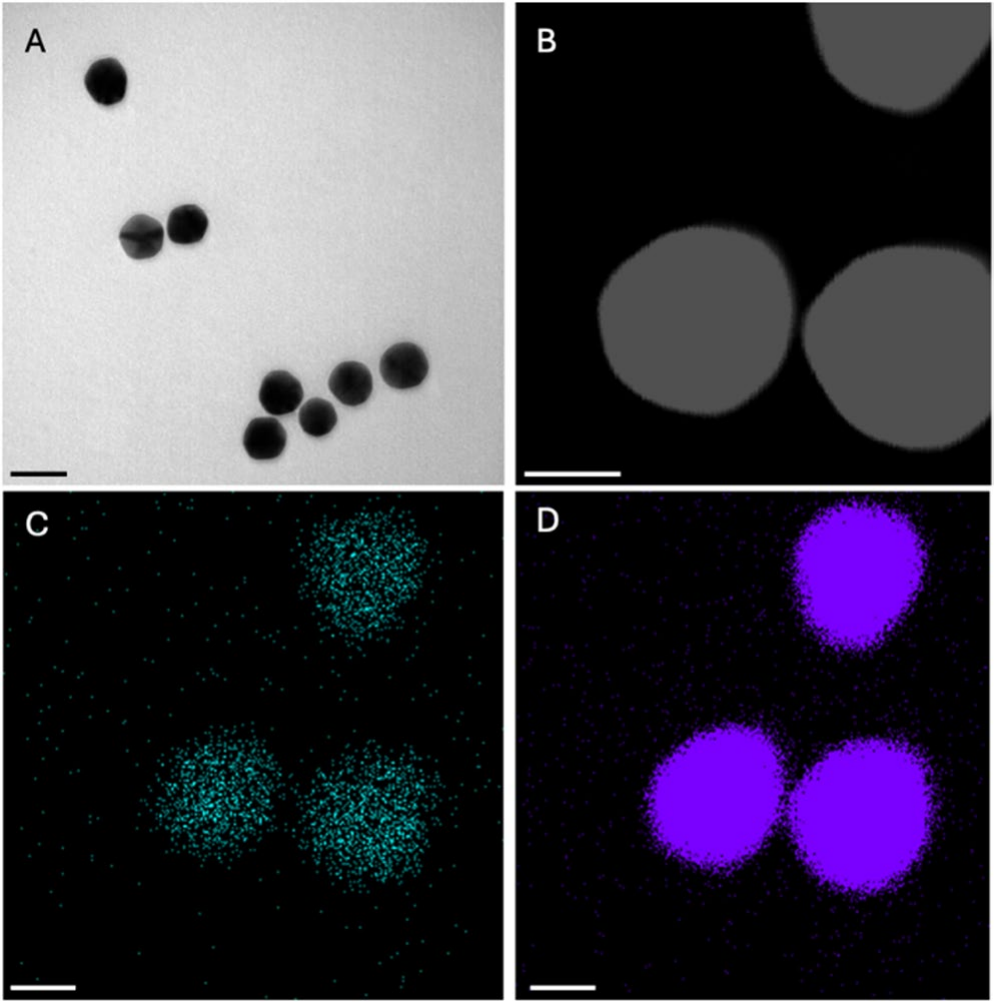
(**A**) Representative TEM image of the conjugates. Scale bar corresponds to 50 nm. (**B**) STEM image of the conjugate and EDX mapping of (**C**) I and (**D**) Au. Scale bar in (B), (C) and (D) corresponds to 20 nm

Transmission electron microscopy (TEM) and energy-dispersive X-ray spectroscopy combined with scanning transmission electron microscopy (EDX-STEM) analysis were used to study the morphology of the conjugates and to confirm the presence of iodine species in them. These techniques allowed direct visualization of the structure of the conjugates and identification of the regions where iodine was present, demonstrating its interaction with the starch and the functionalized nanoparticles (Fig. 3). This confirmed the uniform distribution of the starch-iodine complex in the particles and the absence of aggregation. The EDX spectrum of these particles is shown in Figure S3A.

The hydrodynamic size and ζ potential of AuNP conjugates blocked with iodine-doped starch and BSA were determined by DLS and electrophoretic light scattering (ELS) measurements, respectively. With these values, bioconjugation was confirmed by comparing the hydrodynamic diameter before and after the conjugation reaction. The results obtained for each condition are summarized in Table 1.

**Table 1:** DLS and ELS measurements (ζ average, PdI and ζ potential) of the bare Au NPs and the conjugates blocked with BSA and iodine-doped starch

	Z-average nm	PdI	ζ potential mV
Bare Au NPs	45 ± 1	0.157	−32.2 ± 0.9
Au-αCD63 blocked with BSA conjugate	60 ± 1	0.146	−5.6 ± 0.1
Au-αCD63 blocked with iodine-doped starch conjugate	65.1 ± 0.1	0.167	−8.2 ± 0.2

As can be seen, the difference in size between the bare commercial nanoparticles and both conjugates showed that the conjugation process was successful in both cases (Fig S3B). No significant differences in size, polydispersity or ζ potential were observed between the conjugates blocked with iodine-doped starch and BSA.

Absorbance measurements of gold nanoparticles were used to follow the adsorption of antibodies and proteins or polymers on their surface. A strong absorbance peak between 460 and 560 nm was obtained due to the LSPR of colloidal gold, and its λ_max_ varied depending on the size of the nanoparticles. The maximum wavelength of AuNPs was 530 nm. A redshift to a λ_max_ of 533 nm was observed when the conjugation reaction was performed, blocking with both BSA and iodine-doped starch (Fig S3C). This indicated a change in the refractive index of the nanoparticles due to the organic layer on the nanoparticle surface.

### Electrochemical detection of the nanoparticles

The standard particles for LFIA are AuNPs, but for electrochemical quantification they would have to be dissolved with strong acids or enzymatically digested; hence the need to look for non-hazardous chemical alternatives that can be used by the end-user at home. With the proposed iodine-doped starch-blocked gold particles we get a biosensor that, by integrating the electrochemical probe directly into the LFIA design, would eliminate the need to add external reagents, simplifying the protocol and reducing potential interferences.

The electrochemistry of gold in the presence of iodine is complex due to the similarity between the oxidation potentials of the two species, resulting in overlapping reactions. Gold can be oxidized to form species such as AuI_2_^−^ (E^0^ = 0.578 V) and AuI_4_^−^ (E^0^ = 0.560 V), while iodine participates in parallel reactions such as triiodide formation (E^0^ = 0.535 V) and oxidation (E^0^ = 0.533 V). These reactions compete within the same potential window, making it difficult to distinguish between the individual processes. In addition, iodine species can stabilize the oxidized states of gold, forming complexes that alter the redox behavior. This interaction highlights the highly interdependent nature of the processes in this system [30].

To optimize the fixed potential for coulometry, the following were dispensed separately onto a nitrocellulose membrane, so that it could be seen how the matrix affected the signal: bare Au NPs, bulk iodine-doped starch, and iodine-doped starch-functionalized Au NPs. Then, a cyclic voltammetry (initial potential, E_i_ = 0 V, final potential, E_f_ = 0.8 V, scan rate = 50 mV/s and step potential = 2 mV) was performed for each of them in triplicate (graph not shown). The CV corresponding to the dispensed iodine-starch showed an oxidation peak at 0.34 V. Therefore, a coulometry at 0.45 V would guarantee maximum intensity without losing sensitivity or reproducibility. For the functionalized Au NPs, this peak shifts to 0.43 V because this complex oxidizes at more positive potentials, as previously explained. However, potentials above 0.5 V would result in capacitive currents greater than those of the bare Au NPs, resulting in a loss of sensitivity. For this reason, the fixed potential selected for subsequent coulometries was 0.45 V.

The influence of the area of the working electrode on the coupling of the test line was also analyzed, so we worked with a standard SPE with a circular WE (with a pseudo-referential silver RE) and a customized electrode whose WE had the same shape and size as the test line (with a screen-printed carbon RE) (Fig. S4). With RE of different materials, cyclic voltammetry were performed to identify the redox process (initial potential, E_i_ = −0.5 V, final potential, E_f_ = 0.6 V, scan rate = 50 mV/s and step potential = 2 mV) and in the case of the circular electrode as aforementioned, it was at 0.45 V, while in the case of the line electrode it appeared at 0.62 V. However, the big difference between the two is the peak intensity, 0.349 µA for the circular electrode, and 0.048 µA for the line electrode, a seven-fold decrease. This suggested that although the line electrode was matched the shape of the test line in the strips, the critical factor in this case was the electroactive surface area (ECSA). This was estimated following the Randles-Sevick theory for quasi-reversible systems, obtaining an ECSA of 14.6 mm^2^ for the circular one, 2.7-fold higher than the line one, with 5.37 mm^2^.

### Analytical performance of the eLFIA for EVs quantification

EVs have attracted significant interest due to their potential as biomarkers in several diseases. However, their study is limited by the lack of standardized isolation protocols. While ultracentrifugation and size exclusion chromatography are commonly used methods, they are both time-consuming and expensive. In this study, precipitation methods were used to isolate EVs from plasma, providing a more accessible and faster alternative to these traditional techniques. However, it is important to acknowledge that precipitation methods can compromise the purity of EV fractions by co-precipitating other plasma components. Despite these known limitations, our previous research [31] has shown that such impurities do not affect LFIA performance, which incorporates a separation step when the sample flows through the membrane. This indicates that the eLFIA developed in this work would still be effective. In addition, the design of the eLFIA system, which incorporates a sandwich format with distinct capture and detection antibodies, ensures high specificity for EV identification.

Regarding the sensitivity of the developed eLFIA, it was assessed by measuring different ranges of EV concentrations.

As a first approximation, the range of concentrations previously detected [24] visually in the literature was taken, but in this case we could simultaneously detect EVs visually and electrochemically with a variation of two orders of magnitude in the concentration of EVs. In this and subsequent cases, the limit of detection was calculated using the 3SD/slope criterion.

**Fig. 4:**
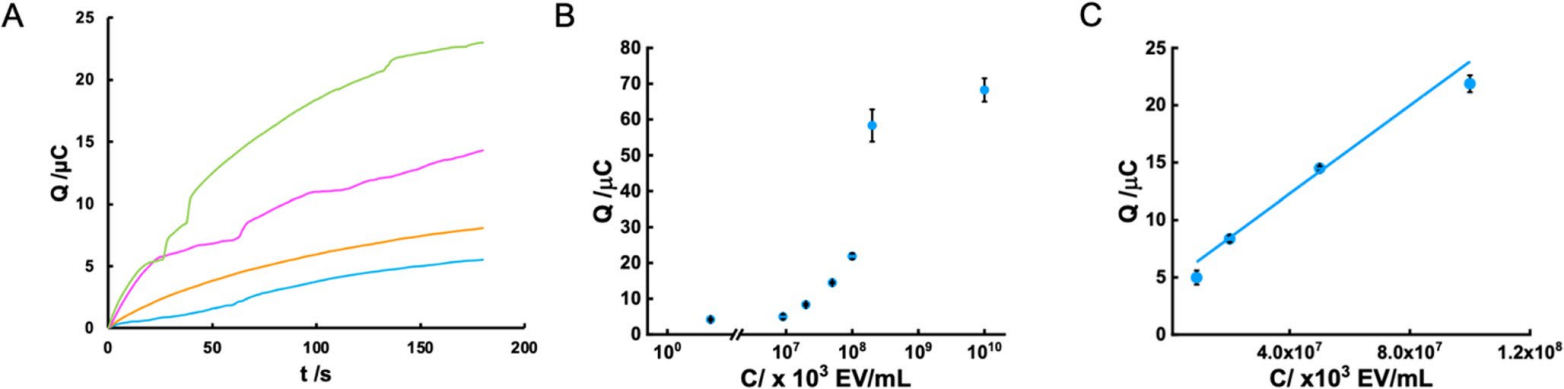
(**A**) Coulometric curves obtained for 9 × 10^9^ (blue), 2 × 10^10^ (orange), 5 × 10^10^ (pink) and 1 × 10^11^ (green), EV/mL. (**B**) Response curve of the developed biosensor. (**C**) Inset: calibration curve for electrochemical detection of EVs with the developed method. Linear fit: Q(µC) = 1.8 × 10^− 10^ [Concentration (EV/mL)] + 4.2

For electrochemical detection, a linear correlation was obtained between the analytical signal and the concentration of EVs over the dynamic range of 9 × 10^6^ to 100 × 10^6^; with a R^2^ of 0.9913, a LOD of 1.15 × 10^10^ EV/mL, corresponding to a concentration of 19 pM, and a RSD of 5.1%. (Fig. 4)

It is also interesting to compare this detection with visual detection (Figure S5). For this purpose, the signals obtained by the two methods (charge in the electrochemical case and optical density in the visual case) were normalized for the concentration range studied. Both methods showed a linear response, although the dynamic range of the concentrations was reduced in the visual reading. It can also be observed that the error in the electrochemical method is higher at the lowest EV concentration, which is expected when the analyte concentration is close to the limit of quantification (LOQ) of the method and the signal-to-noise ratio decreases, whereas the error in the visual method remains more constant. This is due to the matrix effect of the nitrocellulose membrane, which contributes to light scattering in the visual method, but has little effect on charge transfer in the electrochemical method [20]. Finally, the correlation between the two approaches was evaluated by measuring four strips with both techniques, obtaining a good correlation coefficient of 0.9923.

**Fig. 5:**
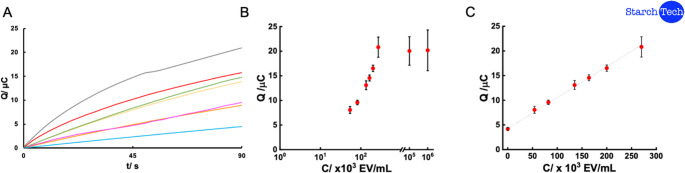
(**A**) Coulometric curves obtained for 0 (blue), 54 (orange), 82 (pink), 135 (yellow), 164 (green), 200 (red) and 270 (grey) x 10^3^ EV/mL. (**B**) Response of the biosensor in low concentration ranges. Inset: linear response from 0 to 2.7 × 10^5^ EV/mL for the developed eLFIA for quantification of EVs

In order to explore the sensitivity and detection limit of the biosensor at lower concentration level, a linear calibration was obtained for the concentration range 0 to 2.7 × 10^5^ EV/mL, where there was not visual detection. This linear response (Fig. 5) was fitted to the Eq. 1, with a linear regression coefficient of 0.9981. This resulted in a LOD (calculated following the 3SD/m criteria) of 9 × 10^3^ EV/mL, equivalent to 15 aM and a LOQ (10SD/m criteria) of 3 × 10^4^ EV/mL, 50 aM. It is important as well to note that this method showed a high reproducibility since its RSD was 6.34%.

1 $$Q\;\left(\mu C\right)=6.1\times10^{-5}\left[Concentration\;\left(EV/mL\right)\right]+4.6$$

It is also interesting to note that the charge values obtained for both linear concentration ranges are quite similar (see Fig. S6). In a set of complementary experiments (not shown), test strips containing Au NPs at concentrations ranging from 0 to 10^14^ particles/mL were measured, showing an increase in charge up to the point at which the test line became optically detectable. This can be explained by the three-dimensional structure of the membrane: the electrochemical probe can access the electrode until the test line becomes saturated and visible to the naked eye. Beyond this point, only the iodine present on the surface of the membrane contributes to the signal. As the number of Au NPs on the surface increases proportionally to the analyte concentration, the measured charge starts to increase again, giving rise to the second linear range.

With the implemented detection method, we demonstrate that the developed biosensor is able to detect and quantify EVs with remarkable versatility. On the one hand, the device allows working in concentration ranges lower to those of established analytical methods such as NTA [32]. On the other hand, it is characterized by exceptional sensitivity, achieving detection at ultra-low levels down to 9 × 10^3^ EV/mL, positioning it as a highly efficient tool for the analysis of EVs in contexts where the detection of minimal concentrations is critical. To place the analytical performance of the developed biosensor in its context, a summary of previously reported LFIA for EVs detection is shown in Table 2, comparing their analytical performance with the eLFIA proposed in this work.

**Table 2:** Comparison of the main characteristics of the developed eLFIA with other LFIAs for the detection and quantification of EVs

Detection strategy for LFIA	Target EVs	Detection nanolabel	Readout device	Sample matrix	LOD EV/mL	Assay time min	Ref
Fluorescent	Biotinylated EV membrane	Fluorescent nanoprobes	Fluorescence reader (desktop)	Buffer (PBS) and human saliva	2 × 10^6^	20	[33]
Fluorescent	CD63+ (universal EVs)	Encapsulated quantum dots	Fluorescent reader (desktop)	Human foreskin fibroblast	1.2 × 10^5^	10	[34]
Fluorescent	CD9+-CD81+ (universal EVs) and EpCAM+-CD81+ (tumor-derived EVs)	Europium NPs	Fluorescence reader (desktop)	Human plasma	2.4 × 10^5^	15	[35]
SERS	CD9+, CD81+ (universal EVs)	Au–Ag nanoshuttles	Raman spectrometer (desktop)	Human plasma	7 × 10^3^	15	[36]
Luminescent	CD63+ (universal EVs)	Upconversion NPs (UCNPs)	Upconversion reader (desktop)	Urine	4 × 10^7^	40	[37]
Electrochemical	CD63+, CD9+ (universal EVs)	Iodine-starch functionalized Au NPs	Potentiostat (portable)	Human plasma	9 × 10^3^	23	This work

The majority of reported LFIA rely on optical detection strategies, such as fluorescence, luminescence or surface-enhanced Raman scattering (SERS). This is the first eLFIA for EV detection. Only one other LFIA, based on SERS detection, has achieved a slightly lower LOD (7 × 10³ EVs/mL), which is in the same order of magnitude as that reported in this study. However, eLFIA using electrochemical detection offer important practical advantages. They can be used with hand-held readers that do not compromise sensitivity. In contrast, even portable SERS-based LFIA require relatively bulky and expensive readers [38, 39], whose signal readout can be interfered with by matrix elements such as proteins or other organic compounds [40]. However, most eLFIAs report similar LODs in buffers and complex samples [41]. These results highlight the potential of the biosensor as a promising and complementary alternative to traditional techniques in clinical settings.

### Application of the eLFIA in human plasma

To validate the feasibility of the designed eLFIA for EV detection in real biological samples, we tested spiked concentrations of the isolated EVs in depleted human plasma by this eLFIA.

As shown in Table 3, recovery rates were among 94 and 101% with low RSD values. At its core, these results indicate that the eLFIA has remarkable accuracy and precision, demonstrating the potential of the developed sensor for the determination of EVs in real biological samples.

**Table 3:** Evaluation of the performance of the e-LFIA for the detection of different concentrations of EVs in human plasma (*n* = 3)

Analyte	Spiked EV/mL	Q µC	Quantified EV/mL	Recovery %	RSD %
Extracellular vesicles	1 × 10^5^	10.5 ± 0.1	9.8 × 10^4^	98	2.20
	1.52 × 10^5^	13.8 ± 0.4	1.53 × 10^5^	101	4.05
	2 × 10^6^	16.0 ± 0.6	1.88 × 10^5^	94	5.04

These results demonstrate a limited matrix effect, which can be attributed to the sandwich configuration of the eLFIA. This configuration requires dual immunorecognition of EVs’ tetraspanins (CD9 and CD63). Therefore, only EVs would be retained on the test line, and the contribution of soluble proteins or circulating tetraspanins would be negligible.

## Conclusions

This study presented a novel biosensor based on an eLFIA for the detection and quantification of EVs. This device was characterized by its ability of detecting ultra-low concentrations down to 9000 EVs/mL (9 EV/µL) and covering ranges comparable to those of traditional techniques. The integration of iodine-doped starch as a functional material not only maintained the stability of the nanoparticles and reduced non-specific interactions, but also allowed the incorporation of an electrochemical probe, simplifying the protocol and avoiding the use of additional reagents. Validation on real biological samples demonstrated high precision and accuracy, with recovery rates above 94%, confirming its applicability in clinical settings. These results position the biosensor as a versatile and efficient tool, capable of complementing and in some aspects surpassing traditional methods for EV detection with the potential to be adapted to any potential biomarker of interest in EVs and can shed light on the diagnosis and monitoring of various diseases. The technology has been protected with an international patent, as Starch Tech™.

## Supplementary Information

Below is the link to the electronic supplementary material.

Supplementary Material 1 (DOCX 1.20 MB)

## Data Availability

The data that support the findings of this study are not openly available due to reasons of sensitivity and are available from the corresponding author upon reasonable request.

## References

[1] Welsh JA, Goberdhan DCI, O’Driscoll L et al (2024) Minimal information for studies of extracellular vesicles (MISEV2023): From basic to advanced approaches. J Extracell Vesicle 13:e12404. 10.1002/jev2.12404 10.1002/jev2.12404 PMC10850029 38326288

[2] Van Niel G, D’Angelo G, Raposo G (2018) Shedding light on the cell biology of extracellular vesicles. Nat Rev Mol Cell Biol 19:213–228. 10.1038/nrm.2017.125 29339798 10.1038/nrm.2017.125

[3] Raposo G, Stahl PD (2019) Extracellular vesicles: a new communication paradigm? Nat Rev Mol Cell Biol 20:509–510. 10.1038/s41580-019-0158-7 31324871 10.1038/s41580-019-0158-7

[4] Xu R, Rai A, Chen M et al (2018) Extracellular vesicles in cancer — implications for future improvements in cancer care. Nat Rev Clin Oncol 15:617–638. 10.1038/s41571-018-0036-9 29795272 10.1038/s41571-018-0036-9

[5] Möller A, Lobb RJ (2020) The evolving translational potential of small extracellular vesicles in cancer. Nat Rev Cancer 20:697–709. 10.1038/s41568-020-00299-w 32958932 10.1038/s41568-020-00299-w

[6] Dickhout A, Koenen RR (2018) Extracellular vesicles as biomarkers in cardiovascular disease; chances and risks. Front Cardiovasc Med 5:113. 10.3389/fcvm.2018.00113 30186839 10.3389/fcvm.2018.00113 PMC6113364

[7] Shao H, Im H, Castro CM et al (2018) New technologies for analysis of extracellular vesicles. Chem Rev 118:1917–1950. 10.1021/acs.chemrev.7b00534 29384376 10.1021/acs.chemrev.7b00534 PMC6029891

[8] Erdbrügger U, Lannigan J (2016) Analytical challenges of extracellular vesicle detection: a comparison of different techniques. Cytometry A 89:123–134. 10.1002/cyto.a.22795 26651033 10.1002/cyto.a.22795

[9] Wang Y, Ali MA, Chow EKC et al (2018) An optofluidic metasurface for lateral flow-through detection of breast cancer biomarker. Biosens Bioelectron 107:224–229. 10.1016/j.bios.2018.02.038 29475186 10.1016/j.bios.2018.02.038

[10] Vaz R, Serrano VM, Castaño-Guerrero Y et al (2022) Breaking the classics: Next-generation biosensors for the isolation, profiling and detection of extracellular vesicles. Biosens Bioelectronics: X 10:100115. 10.1016/j.biosx.2022.100115

[11] Dutta G, Lillehoj PB (2017) An ultrasensitive enzyme-free electrochemical immunosensor based on redox cycling amplification using methylene blue. Analyst 142:3492–3499. 10.1039/C7AN00789B 28831485 10.1039/c7an00789b PMC5600201

[12] Dutta G, Kim S, Park S, Yang H (2014) Washing-Free Heterogeneous Immunosensor Using Proximity-Dependent Electron Mediation between an Enzyme Label and an Electrode. Anal Chem 86:4589–4595. 10.1021/ac5006487 24758236 10.1021/ac5006487

[13] Dutta G, Park S, Singh A et al (2015) Low-Interference Washing-Free Electrochemical Immunosensor Using Glycerol-3-phosphate Dehydrogenase as an Enzyme Label. Anal Chem 87:3574–3578. 10.1021/ac504485a 25751001 10.1021/ac504485a

[14] Serrano-Pertierra E, Oliveira-Rodríguez M, Matos M et al (2020) Extracellular Vesicles: Current Analytical Techniques for Detection and Quantification. Biomolecules 10:824. 10.3390/biom10060824 32481493 10.3390/biom10060824 PMC7357140

[15] Ying X, Fu W, Zhu L et al (2024) Electrochemical Lateral Flow Immunoassay with Built-In Electrodes for Ultrasensitive and Wireless Detection of Inflammatory Biomarkers. Anal Chem 96:10630–10638. 10.1021/acs.analchem.4c01224 38912708 10.1021/acs.analchem.4c01224

[16] Mao X, Baloda M, Gurung AS et al (2008) Multiplex electrochemical immunoassay using gold nanoparticle probes and immunochromatographic strips. Electrochem Commun 10:1636–1640. 10.1016/j.elecom.2008.08.032

[17] Nandhakumar P, Muñoz San Martín C, Arévalo B et al (2023) Redox cycling amplified electrochemical lateral-flow immunoassay: toward decentralized sensitive insulin detection. ACS Sens 8:3892–3901. 10.1021/acssensors.3c01445 37734056 10.1021/acssensors.3c01445

[18] Thobhani S, Attree S, Boyd R et al (2010) Bioconjugation and characterisation of gold colloid-labelled proteins. J Immunol Methods 356:60–69. 10.1016/j.jim.2010.02.007 20188107 10.1016/j.jim.2010.02.007

[19] Rundle RE, Foster JF, Baldwin RR (1944) On the nature of the starch—iodine complex^1^. J Am Chem Soc 66:2116–2120. 10.1021/ja01240a031

[20] Blickenstorfer Y, Jirasko V, Tanno A et al (2024) Iodide based electrochemical gold quantification method for lateral flow assays. Biosens Bioelectron 262:116524. 10.1016/j.bios.2024.116524 38971036 10.1016/j.bios.2024.116524

[21] Marefati A, Wiege B, Haase NU et al (2017) Pickering emulsifiers based on hydrophobically modified small granular starches – Part I: manufacturing and physico-chemical characterization. Carbohydr Polym 175:473–483. 10.1016/j.carbpol.2017.07.044 28917891 10.1016/j.carbpol.2017.07.044

[22] María Carmen Blanco-López, María Matos-González, Gemma Gutiérrez-Cervelló, et al et al (2025) Nanoparticles comprising a starch-iodine complex and their use for immunodetection. European Patent: EP24382448. Priority date: 24/04/2024. PCT/EP2025/060986 (23/04/2025)

[23] Oliveira-Rodríguez M, López‐Cobo S, Reyburn HT et al (2016) Development of a rapid lateral flow immunoassay test for detection of exosomes previously enriched from cell culture medium and body fluids. J Extracell Vesicles 5:31803. 10.3402/jev.v5.31803 27527605 10.3402/jev.v5.31803 PMC4985618

[24] Oliveira-Rodríguez M, Serrano-Pertierra E, García AC et al (2017) Point-of-care detection of extracellular vesicles: sensitivity optimization and multiple-target detection. Biosens Bioelectron 87:38–45. 10.1016/j.bios.2016.08.001 27517736 10.1016/j.bios.2016.08.001

[25] Jazayeri MH, Amani H, Pourfatollah AA et al (2016) Various methods of gold nanoparticles (GNPs) conjugation to antibodies. Sens Bio-Sens Res 9:17–22. 10.1016/j.sbsr.2016.04.002

[26] Morán D, Gutiérrez G, Blanco-López MC et al (2021) Synthesis of starch nanoparticles and their applications for bioactive compound encapsulation. Appl Sci 11:4547. 10.3390/app11104547

[27] Tomasik P, Schilling CH (1998) Complexes of Starch with Inorganic Guests. Advances in Carbohydrate Chemistry and Biochemistry. Elsevier, pp 263–343

[28] Li G, Hemar Y, Zhu F (2021) Relationships between supramolecular organization and amylopectin fine structure of quinoa starch. Food Hydrocolloids 117:106685. 10.1016/j.foodhyd.2021.106685

[29] Wangoo N, Bhasin KK, Mehta SK, Suri CR (2008) Synthesis and capping of water-dispersed gold nanoparticles by an amino acid: Bioconjugation and binding studies. J Colloid Interface Sci 323:247–254. 10.1016/j.jcis.2008.04.043 18486946 10.1016/j.jcis.2008.04.043

[30] Qi PH, Hiskey JB (1993) Electrochemical behavior of gold in iodide solutions. Hydrometallurgy 32:161–179. 10.1016/0304-386X(93)90021-5

[31] Serrano-Pertierra E, Oliveira-Rodríguez M, Rivas M et al (2019) Characterization of plasma-derived extracellular vesicles isolated by different methods: a comparison study. Bioengineering 6:8. 10.3390/bioengineering6010008 30658418 10.3390/bioengineering6010008 PMC6466225

[32] Malvern Panalytical In: NanoSight Pro. https://www.malvernpanalytical.com/en/products/product-range/nanosight-range/nanosight-pro?utm_source=google&utm_medium=cpc&utm_campaign=EN%20-%20WESEU%20-%20Search%20(ROAS)&utm_term=nanosight%20pro&utm_content=EN%20-%20Product%20-%20NanoSight%20Pro&gad_source=1&gad_campaignid=20878026644&gbraid=0AAAAAD4oRF38voBLQggcQgtcc__5JyA8q&gclid=CjwKCAjwpOfHBhAxEiwAm1SwEhW5jmMm_sGcfbQf1AJWg8dRh_thuuxN61EYQ0KivY4AQxBwdqCp-hoCKowQAvD_BwE. Accessed 23 Oct 2025

[33] Dong D, Zhu L, Hu J et al (2019) Simple and rapid extracellular vesicles quantification via membrane biotinylation strategy coupled with fluorescent nanospheres-based lateral flow assay. Talanta 200:408–414. 10.1016/j.talanta.2019.03.069 31036202 10.1016/j.talanta.2019.03.069

[34] Kim H-M, Oh C, An J et al (2021) Multi-quantum dots-embedded silica-encapsulated nanoparticle-based lateral flow assay for highly sensitive exosome detection. Nanomaterials 11:768. 10.3390/nano11030768 33803623 10.3390/nano11030768 PMC8002883

[35] Lu C, Xiao W, Su Y et al (2023) Rapid evaluation of lung adenocarcinoma progression by detecting plasma extracellular vesicles with lateral flow immunoassays. ACS Sens 8:1950–1959. 10.1021/acssensors.2c02706 37195005 10.1021/acssensors.2c02706

[36] Wang M, Wang Y, Wang C et al (2024) Development point-of-care based lateral flow biosensor for the rapid detection of exosomes of subarachnoid hemorrhage patients. J Mater Chem C 12:17159–17169. 10.1039/D4TC02620A

[37] Islam MK, Mahmud I, Ali K et al (2025) High-sensitivity detection of urinary extracellular vesicles with upconverting nanoparticle‐based lateral flow immunoassay. J Extracell Bio 4:e70053. 10.1002/jex2.70053 40625368 10.1002/jex2.70053 PMC12229717

[38] Khlebtsov B, Khlebtsov N (2020) Surface-enhanced raman scattering-based lateral-flow immunoassay. Nanomaterials 10:2228. 10.3390/nano10112228 33182579 10.3390/nano10112228 PMC7696391

[39] Perju A, Wongkaew N (2021) Integrating high-performing electrochemical transducers in lateral flow assay. Anal Bioanal Chem 413:5535–5549. 10.1007/s00216-021-03301-y 33913001 10.1007/s00216-021-03301-y PMC8410735

[40] Heo B, Jung HS (2025) SERS-driven evolution of lateral and vertical flow assays in medical diagnostics. Biosensors 15:573. 10.3390/bios15090573 41002312 10.3390/bios15090573 PMC12467469

[41] Qi M, Fu W, Ying X et al (2025) Microneedle combined with an electrochemical lateral flow immunoassay strip for highly sensitive and wide-range detection of protein biomarkers by increasing the reaction contact area. Anal Chem 97:10638–10645. 10.1021/acs.analchem.5c00420 40356265 10.1021/acs.analchem.5c00420

