# Supplementary material for: Ultrasensitive electrochemical dipstick lateral flow immunoassay for extracellular vesicle quantification using starch-iodine functionalized gold nanoparticles
Source: Mikrochim Acta. 2026 Apr 22;193(5):336. doi: 10.1007/s00604-026-08063-x (PMC13102852; doi:10.1007/s00604-026-08063-x)
Supplement: Supplementary file 1 — Supplementary Material 1 (DOCX 1.20 MB) [file 604_2026_8063_MOESM1_ESM.docx]

**SUPPLEMENTARY MATERIAL**

**Ultrasensitive electrochemical dipstick lateral flow immunoassay for extracellular vesicle quantification using starch-iodine functionalized gold nanoparticles**

**Clara Saweres-Argüelles^a,c^, Alberto Sánchez-Calvo^a^, Esther Serrano-Pertierra^b^, Gemma Gutiérrez^c^, María Matos^c^, María Carmen Blanco-López^a^***

*^a^ Department of Physical and Analytical Chemistry & Institute of Biotechnology of Asturias, University of Oviedo, Faculty of Chemistry, C/ Julián Clavería 8, 33006 Oviedo, Spain*

*^b^ Department of Biochemistry & Institute of Biotechnology of Asturias, University of Oviedo, Santiago Gascón building, C/ Julián Clavería s/n, 33006 Oviedo, Spain*

*^c^ Department of Chemical and Environmental Engineering & Institute of Biotechnology of Asturias, University of Oviedo, Faculty of Chemistry, C/ Julián Clavería 8, 33006 Oviedo, Spain*

***Microchimica Acta***

**Corresponding author:**

*cblanco@uniovi.es (María Carmen Blanco-López)

**ORCID number:**

Clara Saweres-Argüelles: 0009-0004-8542-9902

Alberto Sánchez-Calvo: 0009-0005-0208-8450

Esther Serrano-Pertierra: 0000-0001-8356-858X

Gemma Gutiérrez: 0000-0003-2700-4944

María Matos: 0000-0002-6980-0554

María Carmen Blanco-López: 0000-0002-9776-9013

**TABLE OF CONTENTS**

| **SUPPORTING SECTIONS** | | **Pg.** |
| --- | --- | --- |
| **SM.1** | Reagents and apparatus | **S3** |
| **SM.2** | Characterization of the particles and the conjugates | **S3** |
|  |  |  |
| **SUPPORTING FIGURES** | |  |
| **Figure S1**  **Figure S2**  **Figure S3**  **Figure S4**  **Figure S5**  **Figure S6** | Optimization of the starch-iodine stabilization of Au NPs  Flocculation assay  Characterization of the bioconjugate  Electrodes employed with different geometries  Comparison between visual and electrochemical approaches  Full dose-response of the eLFIA | **S5**  **S6**  **S7**  **S8**  **S9**  **S10** |

**SUPPORTING SECTIONS**

**SM.1 Reagents and apparatus**

Commercial 40 nm gold nanoparticle (AuNP) solution was purchased from BBI solutions (Cardiff, UK). Iodine, Reagent ACS, 99.8% was obtained from Fisher Scientific and native quinoa starch used in this study was provided by Dr. Marilyn Rayner (Lund University, Lund, Sweden) and characterized in previous reports1.

Anti-CD63 mouse monoclonal antibody was purchased from Immunostep (Salamanca, Spain).

As for the components of the strip, the membrane (Hi-Flow™ Plus HF180) and the glass fiber sample pads (GFCP001000) were purchased from Millipore (Darmstadt, Germany), the backing cards (KN-V1080) from Kenoshatapes (Amstelveen, Netherlands) and the absorbent pads from Whatman (Piscataway, USA).

Anti-Mouse IgG, N-hydroxysuccinimide (NHS), 1-ethyl-3-[3-dimethylaminopropyl]-carbodiimide hydrochloride (EDC), bovine serum albumin (BSA), sodium phosphate monobasic and sodium phosphate dibasic were purchased from Sigma-Aldrich (Darmstadt, Germany). Also, HEPES was acquired from Fisher Scientific.

ExoQuick precipitation reagent was acquired from System Biosciences, Palo Alto, CA.

With these last reagents, phosphate buffer (PB) and HEPES buffer were prepared both at a concentration 10 mM at pH 7.4 and filtered for their subsequent uses.

In terms of instrumentation, an IsoFlow reagent dispensing system (ImageneTechnology, USA) was used to dispense the detection lines and the strips were cut using a Fellowes Gamma (Spain) guillotine.

Regarding the electrochemical measurements, screen-printed electrode cards (DRP-110) with a carbon ink working and auxiliary electrode and a silver pseudoreference electrode and personalized SPCE (DRP-P-111-UO) with the three carbon ink electrodes were obtained from Metrohm Dropsens (Spain). The connections with the electrodes are made with a DSC connector (DRP-DSC) from the same company. The measurements were carried out using a µStat-I 400s potentiostat, controlled by Dropview 8400 software.

**SM.2 Characterization of the particles and conjugates**

Dynamic light scattering (DLS) and electrophoretic light scattering (ELS) were used to measure the hydrodynamic size and ξ potential of the nanoparticles, respectively, using a Zetasizer Nano ZS instrument (Malvern Instruments Ltd, Malvern, UK).

The particle morphology and aggregation structure were determined by TEM. An aliquot of an aqueous suspension of the samples was placed on a transparent carbon sheet with a copper grid support and analyzed using a MET JEOL-2000 EXII TEM (Saint-Herblain, France). Using the same sample preparation, high-resolution transmission electron microscopy (HR-TEM) studies were performed on a JEOL JEM-2100F transmission electron microscope operating at an accelerating voltage of 200 kV. This TEM is also equipped with a scanning transmission electron microscope (STEM) control unit (Gatan) and an energy dispersive X-ray (EDX) detector (Oxford Instruments, X-Max SDD 80 mm2), and this microscope was used to perform HR-TEM and EDX-STEM analysis (line scan and area mapping).

The ultraviolet-visible spectrum were also recorded to verify both conjugation and agglomeration of the nanoparticles. For this, absorbance measurements of the aqueous suspensions of both the commercial particles and the conjugates were performed in a Cary 60 UV-Vis spectrophotometer from Agilent Technologies (Palo Alto, CA, USA).

**SUPPORTING FIGURES**

**
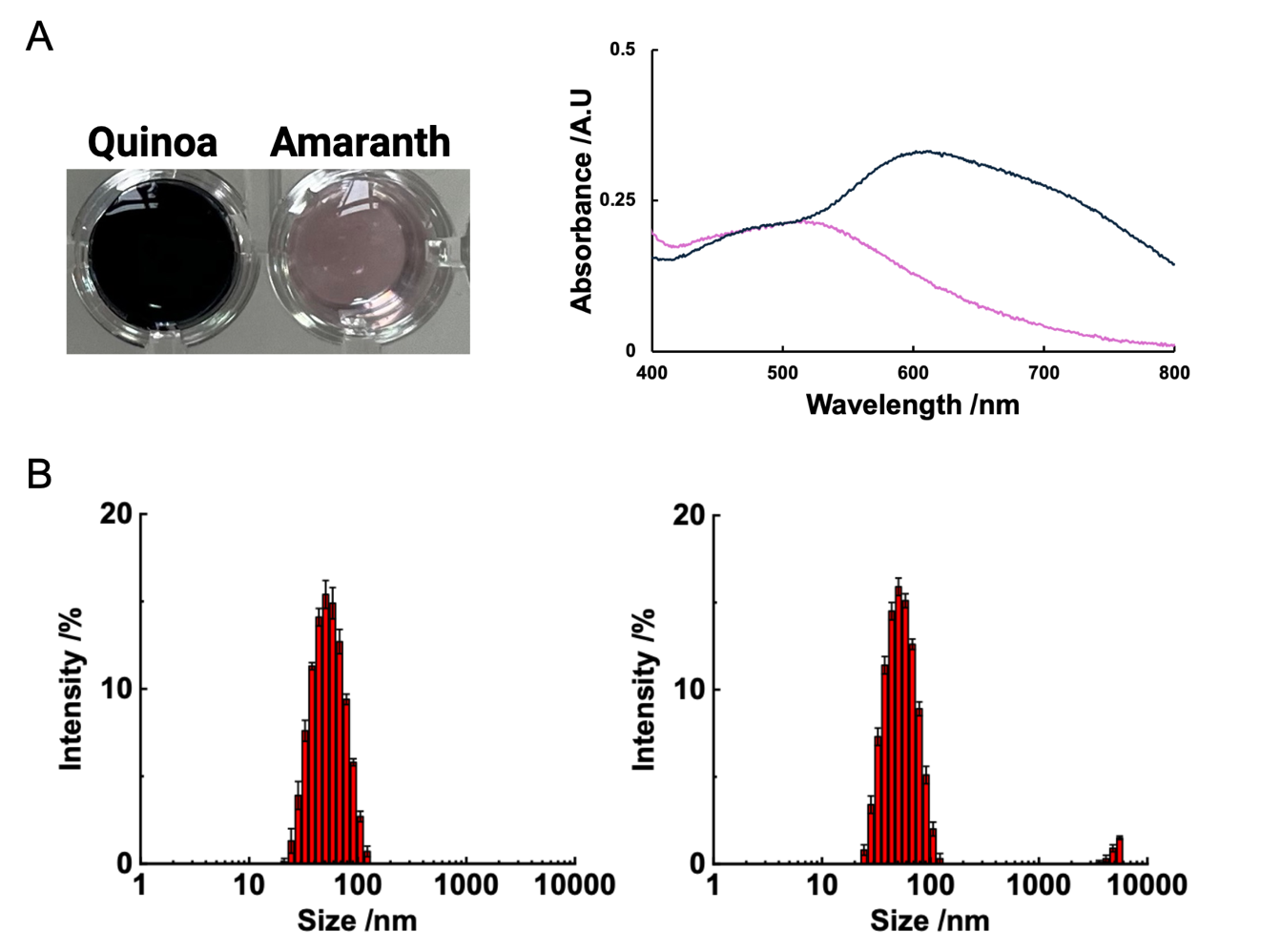
**

**Figure S1.** (**A**) Physical appearance of the starch-iodine complexes with quinoa and amaranth starches and UV-Vis absorption spectra of these complexes with quinoa (blue) and amaranth (pink). (**B**) DLS histogram for Au NPs blocked with 1 (left) and 5 (right) mg/mL of iodine-doped starch.


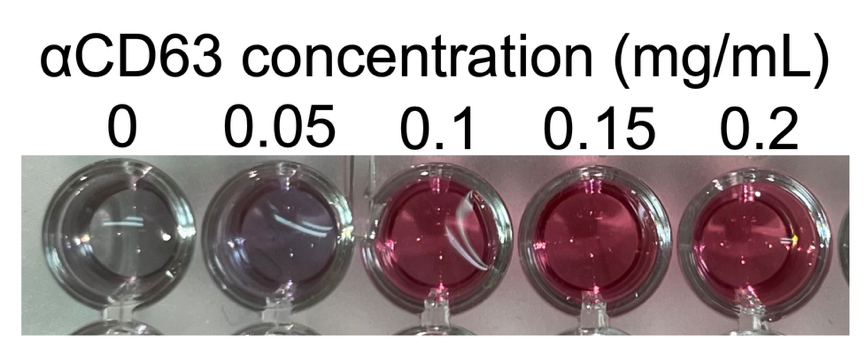


**Figure S2.** Flocculation assay with Au NPs bioconjugated to αCD63 after the addition of a 10% NaCl solution.


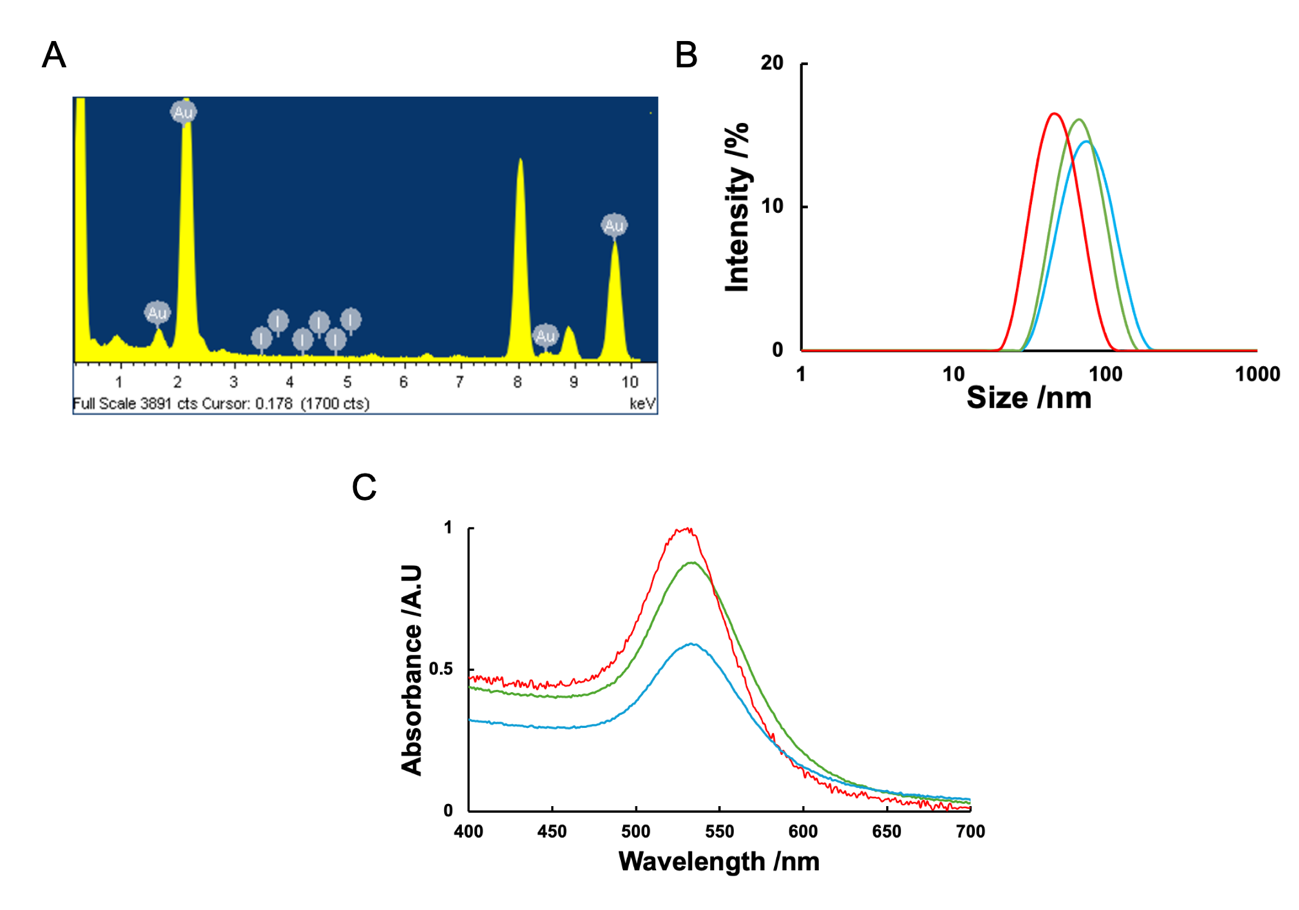


**Figure S3.** (**A**) EDX spectrum of the bioconjugate blocked with iodine-doped starch, (**B**) Hydrodynamic size distribution of bare Au NPs (red), Au-αCD63 blocked with BSA conjugate (green), and Au-αCD63 blocked with iodine-doped starch conjugate (blue) measured by DLS, (**C**) UV-Vis spectra of bare Au NPs (red), Au-αCD63 blocked with BSA conjugate (green), and Au-αCD63 blocked with iodine-doped starch conjugate (blue).

**
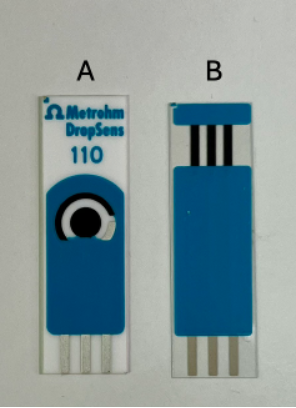
**

**Figure S4.** (**A**) Commercial electrode with carbon ink circular WE and AE and silver pseudoreference and (**B**) Custom electrode with linear carbon ink WE, AE and RE.


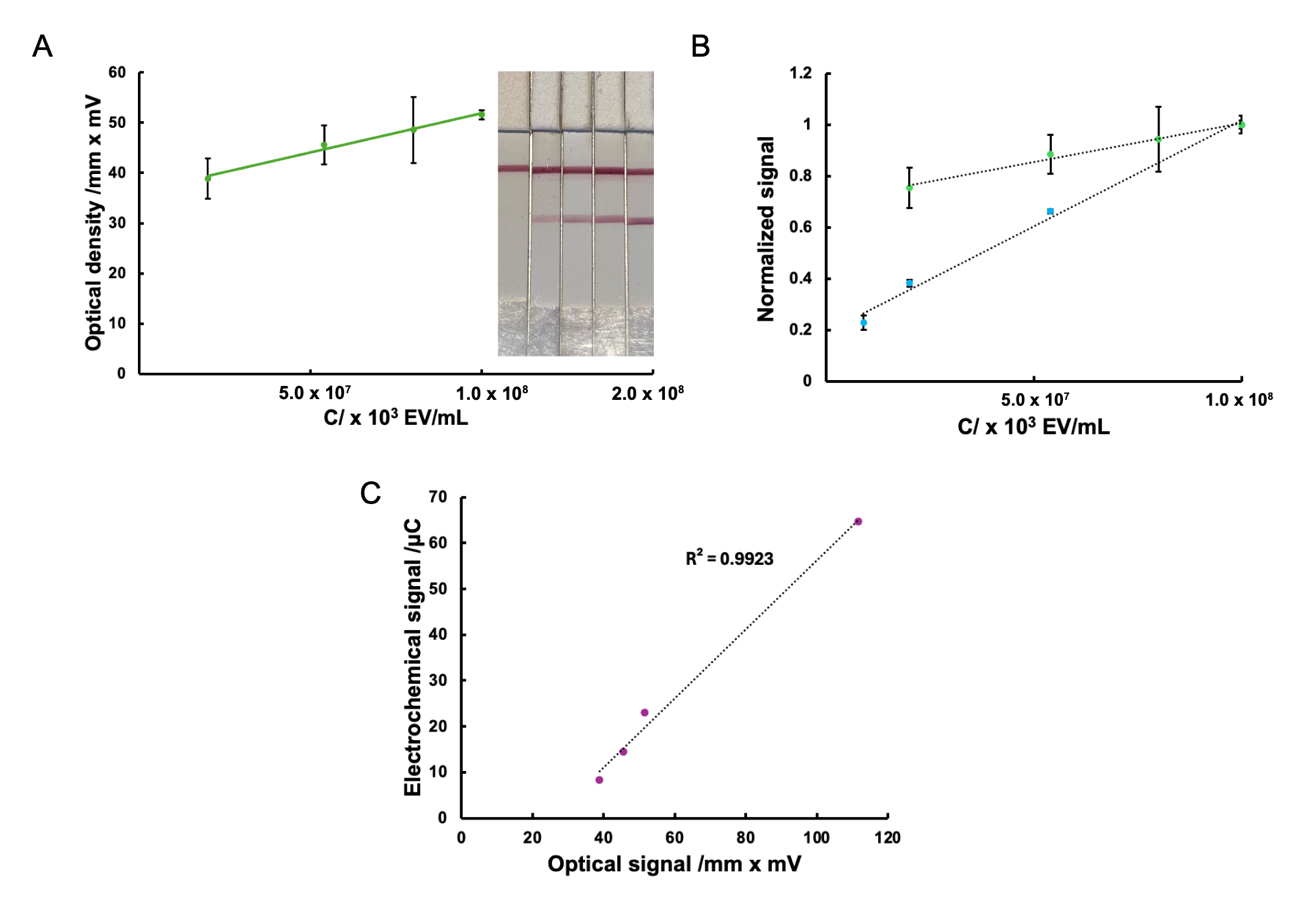


**Figure S5.** (**A**) Calibration curve obtained for 9 x 10^9^-1 x 10^11^ EV/mL along with pictures of the corresponding strips. Linear fit: Q(µC) = 1.5 x 10^-10^ [Concentration (EV/mL)] + 36.2 (**B**) Comparison between visual (in green) and electrochemical (in blue) normalized signals for the same range of concentrations. (**C**) Correlation among the electrochemical and visual signals.

**
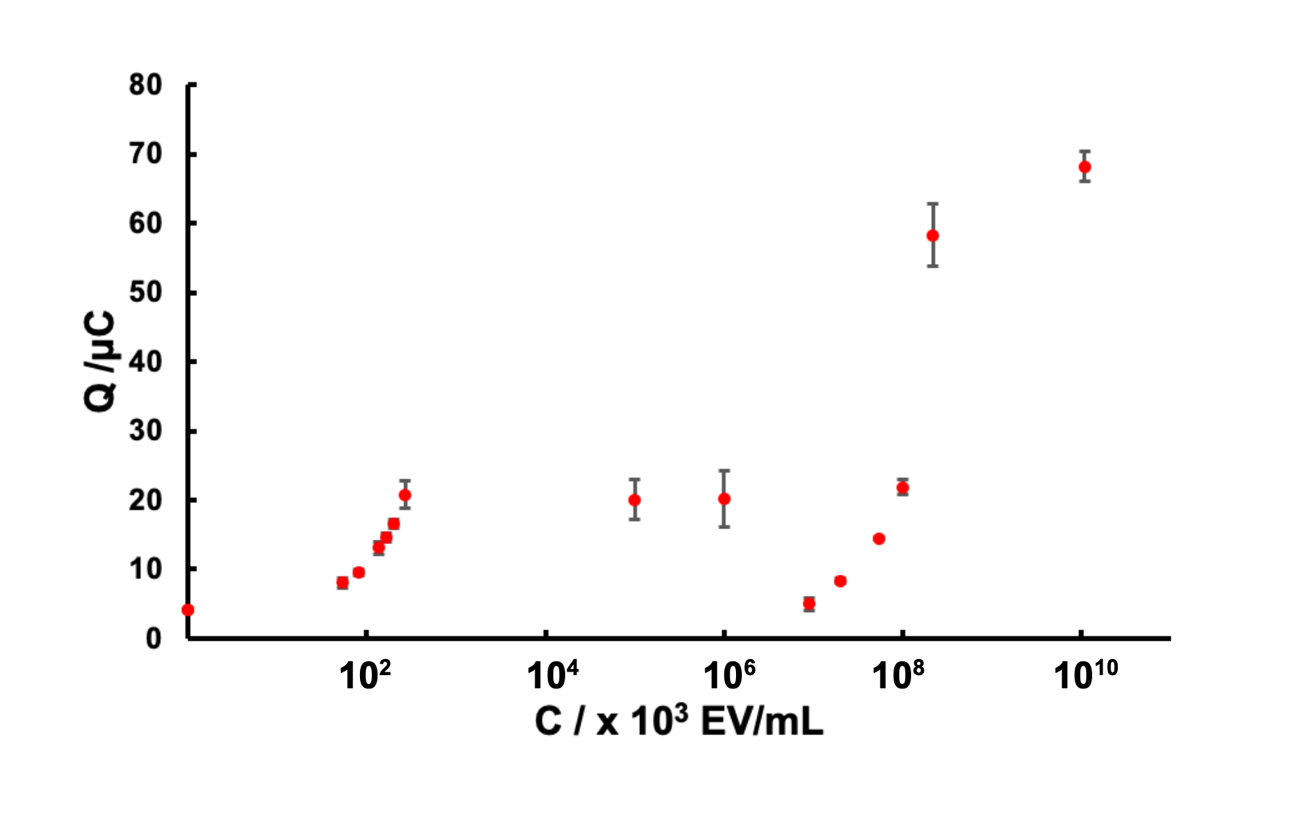
**

**Figure S6.** Dose-response curve for the full range of EV measured concentrations, showing both linear ranges obtained.
